# Laboratory Microprobe X-Ray Fluorescence in Plant Science: Emerging Applications and Case Studies

**DOI:** 10.3389/fpls.2018.01588

**Published:** 2018-11-14

**Authors:** Eduardo S. Rodrigues, Marcos H. F. Gomes, Nádia M. Duran, João G. B. Cassanji, Tatiana N. M. da Cruz, Analder Sant’Anna Neto, Susilaine M. Savassa, Eduardo de Almeida, Hudson W. P. Carvalho

**Affiliations:** ^1^Nuclear Instrumentation Laboratory, Center for Nuclear Energy in Agriculture, University of São Paulo, Piracicaba, Brazil; ^2^Physiology of Trees Laboratory, Department of Forest Science, College of Agriculture Luiz de Queiroz, University of São Paulo, Piracicaba, Brazil

**Keywords:** microprobe XRF, seed priming, phytopathogenic fungi, mineral nutrient uptake, absorption and transport of nutrients, lead phytoextraction, *in vivo* imaging, 2D elemental distribution

## Abstract

*In vivo* and micro chemical analytical methods have the potential to improve our understanding of plant metabolism and development. Benchtop microprobe X-ray fluorescence spectroscopy (μ-XRF) presents a huge potential for facing this challenge. Excitation beams of 30 μm and 1 mm in diameter were employed to address questions in seed technology, phytopathology, plant physiology, and bioremediation. Different elements were analyzed in several situations of agronomic interest: (i) Examples of μ-XRF yielding quantitative maps that reveal the spatial distribution of zinc in common beans (*Phaseolus vulgaris*) primed seeds. (ii) Chemical images daily recorded at a soybean leaf (*Glycine max*) infected by anthracnose showed that phosphorus, sulfur, and calcium trended to concentrate in the disease spot. (iii) *In vivo* measurements at the stem of *P. vulgaris* showed that under root exposure, manganese is absorbed and transported nearly 10-fold faster than iron. (iv) Quantitative maps showed that the lead distribution in a leaf of *Eucalyptus* hybrid was not homogenous, this element accumulated mainly in the leaf border and midrib, the lead hotspots reached up to 13,400 mg lead kg^-1^ fresh tissue weight. These case studies highlight the ability of μ-XRF in performing qualitative and quantitative elemental analysis of fresh and living plant tissues. Thus, it can probe dynamic biological phenomena non-destructively and in real time.

## Introduction

X-ray fluorescence (XRF) spectroscopy is a well-established analytical technique for qualitative and quantitative elemental evaluation. It is a multielemental, simultaneous technique and additionally a non-destructive tool, thus being suitable for *in vivo* plant analysis (Haschke, 2014). Particularly, the energy dispersive X-ray fluorescence microprobe (μ-XRF), as microanalysis technique, allows single point, 1D line and 2D mapping elemental determination in broad range of applications in agricultural and forestry science.

Conversely, other analytical techniques, which permit direct sample analysis, such as laser-induced breakdown spectroscopy (LIBS) and laser ablation inductively coupled plasma mass spectrometry/optical emission spectroscopy (LA-ICP-MS/OES), are destructive techniques. Therefore, they do not allow *in vivo* analysis. Hence, herein we present some μ-XRF cases studies in plant science exploring this unique advantage.

**FIGURE 1:**
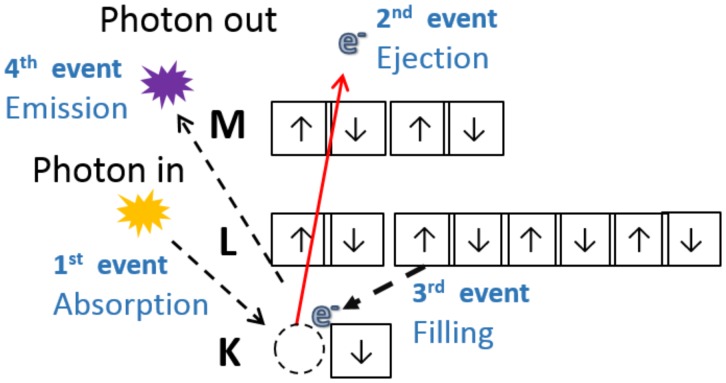
The incoming photon is absorbed by an inner shell electron, the electron is ejected with certain kinetic energy that is equal to the difference between the energy of incoming photon and the binding energy. The departure of the electron excites the atom, then it relaxes while an outer electron fills the hole left in the inner shell. During the relaxation, the atoms can emit heat, another secondary electron (Auger process) or a characteristic photon (XRF).

X-ray fluorescence is the emission of characteristic electromagnetic radiation resulted from a relaxation process. Figure 1 illustrates a series of events that take place during photon induced atomic excitation and further relaxation. Once an X-ray photon impinges upon matter with energy higher than the ionization energy of an inner shell electron, the latter particle may be ejected, producing a vacancy in the corresponding orbital. Subsequently, an electron from an upper orbital fills this vacancy, and the excess of energy can be emitted as a photon. If one ignores screening effects, the energy of this photon is approximately equal to the difference between the energy of the upper and inner orbitals. The emitted energy is characteristic for each chemical element, thus one can use this energy as a fingerprint that allows the elemental identification (Van Grieken and Markowicz, 1993). Moreover, the number of emitted photons is directly proportional to the amount of emitting atoms, thus the XRF peak area yields quantitative information.

There are several types of Energy Dispersive X-ray Fluorescence (EDXRF) systems available (Tsuji et al., 2005; Beckhoff et al., 2007; Margui, 2013). One can highlight the conventional benchtop EDXRF spectrometer, handheld EDXRF (McLaren et al., 2012; Kalcsits, 2016; Guerra et al., 2017) and μ-XRF ones (Beckhoff et al., 2006; Margui, 2013; Haschke, 2014). They can perform analysis of liquids and solids, normally detecting all elements with atomic number above Na in the mg kg^-1^ concentration range (Beckhoff et al., 2006; Margui, 2013; Navas et al., 2016). It is important mentioning that the sensitivity depends also on the chemical element, for example heavier atoms such as Fe or Zn present higher sensitivity, and therefore lower limits of detection (LOD), than P or K.

**FIGURE 2:**
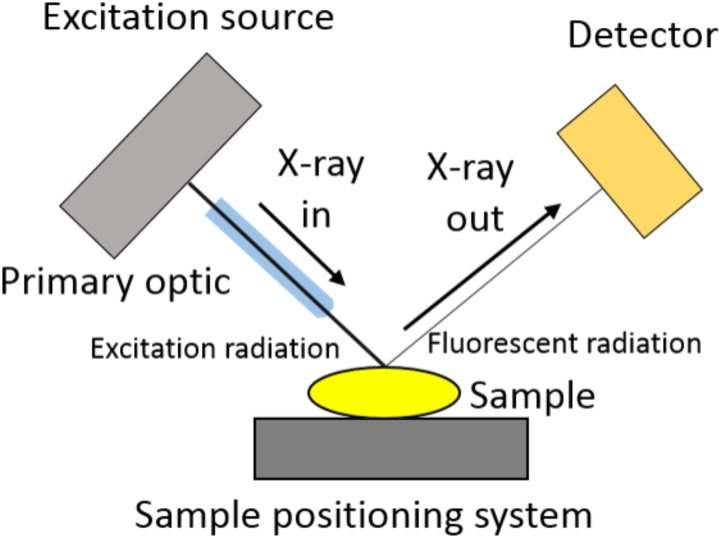
Microprobe X-ray fluorescence spectrometer scheme. The X-ray beam coming from the excitation source is size defined by a focusing or collimating primary optic element. Once it excites the sample placed on a sample holder, the X-ray florescence energy is discriminated and counted by a detector.

The basic design of a μ-XRF spectrometer is presented in Figure 2. A similar μ-XRF equipment, specially designed for the *in vivo* analysis of plants, was built by Fittschen et al. (2017). The X-ray beam, usually produced by the collision of an electron beam against a metallic anode, is shaped and size defined by a primary optic element. This can be a simple collimator, an optical capillary or a focusing mirror. The sample is assembled in a positioning system and the X-ray fluorescence is analyzed by a detector able to discriminate both, photon yield and energy.

X-ray microprobes can be employed as a high throughput analytical system or explored when lateral resolution is required (Tian et al., 2015). In the framework of plant science, μ-XRF presents some complementary features compared to other microprobe and elemental imaging techniques such as scanning electron microscopy-energy dispersive spectroscopy (SEM-EDS) and transmission electron microscopy (TEM-EDS). One can highlight the simplicity of sample preparation for μ-XRF, since differently from SEM and TEM, it does not require conductive coating or thin slicing. Additionally, the μ-XRF LOD are at least one order of magnitude lower than those of SEM-EDS. It happens mainly due to the smaller spectral background found in XRF. Finally, μ-XRF does not require vacuum, although low pressure or He atmosphere can improve the ability of detection for light elements (*Z* < 22). Altogether, these features make μ-XRF an ideal tool for the analysis of fresh vegetal tissues or even *in vivo* plants. Table 1 shows the main μ-XRF advantages and drawbacks.

**Table 1:** Advantages and drawbacks of benchtop microprobe X-ray fluorescence in plant science.

Advantage	Drawback
Minimal sample preparation	mg kg^-1^ limit of detection
Lateral resolution	Matrix effects
*In vivo* analysis (neglected radiation damage in plant – much lower than in a Synchrotron facility)	Low sensitivity for low atomic number element (namely Z < 22)
Multielemental and simultaneous	No detection of some important plant nutrients (e.g., N and B)
Non-destructive (allowing *in vivo* analysis and reanalysis of stored sample)	
Low cost of operation (no gas carrier needed)	
Allowing heterogeneous sample analysis	


One of the major challenges in μ-XRF regards the matrix effects. The probability of the penetration of the excitation X-ray beam, as well as the probability of escape of an X-ray photon emitted by an analyte within the sample, depends on the matrix composition, density, and energies involved in excitation and emission. The XRF photon yield of a given element at a certain concentration in a light atom matrix, carbon for instance, will be higher than the XRF yield for this same element at the same concentration in a heavier matrix such as silicon. Therefore, calibration curves must be built using standards whose matrices are as close as possible to those of the sample. Other possibility consists in calculating the photon attenuation caused by the matrix; this is called fundamental parameter approach. The LOD of μ-XRF in general are several orders of magnitude higher than those provided by LA-ICP-MS.

The matrix effects can be neglected for samples considered “infinitely thin,” it means that the sample thickness does not break the linear relationship between the XRF intensity and the concentration (mass per unit area). In this case, the self-attenuation of the XRF is low enough to be ignored (Van Grieken and Markowicz, 1993; Duran et al., 2017). In case of “intermediate thickness,” i.e., the XRF intensity depends on the sample thickness, the matrix effects can be calculated and corrected (Van Grieken and Markowicz, 1993; Blonski et al., 2007). These two situations will be treated in detail below. There are also samples called “infinitely thick.” In this case, the increasing the sample mass per unity of area does not influence the intensity of the XRF peak. For this type of sample, the quantitative analysis is usually accomplished by external analytical curves, standard addition, fundamental parameters or calibrating the equipment with a set of reference certified material whose matrix is similar to that of the sample.

One of main concerns regarding *in vivo* XRF measurements regards radiation-induced tissue damage. The radiation can damage biological tissues due to photolysis or heating. The dose necessary to cause such damages is ca. 10^7^ Gy (1 Gray = 1 Joule kg^-1^) (Henderson, 1990). There was no evidence of radiation induced damage in the studies reported hereafter.

One the main limitations of XRF in the agronomic context concerns the difficulties involved in the detection of nitrogen and boron. Firstly, the XRF yield of such elements are low since they relaxes mainly via Auger emission (Van Grieken and Markowicz, 1993). Also, the low energy of the XRF photons require the use of vacuum.

Other fact that must be improved regards the lateral resolution, or magnification of XRF imaging. The beam size available in benchtop machines (tens of micrometers wide) (Janssens et al., 2000; Tsuji et al., 2005; Haschke, 2014) are relatively large if compared to synchrotron facilities (from micrometers to nanometers) (Castillo-Michel et al., 2017). This limits the application of benchtop μ-XRF to the investigation of a collection of cells while in synchrotrons a single cell can be mapped.

For infinitely thick and intermediate thickness samples, the elemental spatial correlation can become trick since the probability of photon escape depends on both energy and depth. For example, in a cellulose matrix 50 μm thick and density of 1 g cm^-3^ the transmission of Kα photons from Zn is 94% whereas that of Kα emitted by P is 0.8%. Thus, if a bunch of Zn atoms is beneath a bunch of P atoms, in a 2D projection they may appear in the same region indicating positive spatial correlation while they do not occupy the same volume in the space, this issue is illustrated in the Supplementary Figure 1.

Considering that under realistic conditions plants are exposed to micronutrients at concentration ranging from sub to few mg L^-1^ and normal concentration levels in tissues falls typically in tens of mg kg^-1^, the main challenge concerning *in vivo* studies lies on the improving LOD. A strategy to achieve it could rely on customized equipment carrying optimized primary filters and higher solid angle detection. This latter one can be done by increasing the surface area of detectors (which also increases costs) or allowing experimentalist to tune the distance between sample and detector.

Our group has applied benchtop laboratory μ-XRF in plant science and this manuscript aimed at bringing up a set of μ-XRF methods and procedures which have been developed and used mainly for agronomical and environmental studies. Our goal is to highlight the application μ-XRF for the plant science community. In this paper, we feature the usage of μ-XRF to unravel the spatial distribution of nutrients in primed seeds, the dynamics of elemental redistribution on a fungi infected leaf, the *in vivo* monitoring of competitive uptake of Mn and Fe passing through the stem of common bean and finally the spatial distribution of Pb accumulated in the leaves of *Eucalyptus*.

## Materials and Methods

All experiments were carried out using a benchtop μ-XRF system (Orbis PC EDAX, United States) furnished with a Rh anode with max power rating at 50 kV and 1000 μA. The machine operates with 1 and 2 mm collimators, or 30 μm polycapillary optic. This facility is also equipped with 25 μm Al, 25 μm Ti, 25 μm Ni, 100 μm Rh, 127 μm Nb, and 250 μm Al optional primary filters. The detection was carried out by a 30 mm^2^ silicon drift detector (140 eV FWHM at the 5.9 keV Mn-Kα line). The specific conditions such as X-ray beam size, tube current and voltage, dwell time, and number of points used in each of the examples are shown in Table 2. The pixels produced by the Orbis Vision software were linearly interpolated using Origin Lab 2016, the details on the algorithm can be found at Origin Lab user’s manual.

**Table 2:** Instrumental parameters used in the μ-XRF analysis.

Application	Type of analysis/matrix	Tube voltage (kV)	Tube current (μA)	Primary filter	Vacuum	Beam size	Tissue	Dwell time (s)	Dead time
Seed technology	Map/64 × 50	40	300	25 μm Ni	No	30 μm	Seed	1	<5%
Phytopathology	Map/64 × 50	40	900	none	No	30 μm	Leaf	2	<3%
Mineral nutrition	Single point	45	900	25 μm Ti	No	1 mm	Stem	120	<3%
Bioremediation	Map/32 × 25	40	300	25 μm Ni	Yes	30 μm	Leaf	3	<3%


### Quantitative Zn Mapping in Primed Bean Seed

*Phaseolus vulgaris* (common bean) seeds were soaked for 20 min in a ZnSO_4_.7H_2_O solution at 1,000 mg L^-1^, dried at room temperature for 24 h and carefully cut in the middle using a stainless-steel blade. Part of the seeds had their backs sliced yielding 1–2 mm sections while another part was preserved as shown in Supplementary Figure 2. The split seeds were placed in a sample holder with a polyimide tape with the cotyledon’s inner side exposed for analysis. Supplementary Figure 3 presents the experimental setup used in this analysis (see Table 2 for details on the instrumental parameters).

To build up the quantitative Zn map of the treated seeds, we determined the Zn concentration using equation (1) (Leroux and Mahmud, 1966).

(1) $$C\;(\mathrm{\mu g}\;{\mathrm{cm}}^{-2})=\frac{I_{sample\;above\;threshold(\mathrm{cps})}*Ab_{correction}}{S\;(\mathrm{cps}\;{\mathrm{\mu g}}^{-1}\;{\mathrm{cm}}^{2})}$$

where, *C* is the Zn concentration in the sample (μg cm^-2^), *I_sample above threshold_* is the Zn XRF net intensity emitted by the analyte (cps, counts per second), *Ab_correction_* is the absorption correction factor (dimensionless) detailed in equation (3) and (4), and *S* is the elemental sensitivity for Zn (cps μg^-1^ cm^2^). The threshold (cps) selects the analytical signal suitable for quantitative analysis, it is calculated according to equation 2.

(2) $$threshold(\mathrm{cps})=8.45*\sqrt{\frac{BG_{\left(average)(\mathrm{cps}\right)}}{t(s)}}$$

where, *BG*_(average)_ (cps) corresponds to the average background calculated from ten randomly selected points within the sample and *t*(s) is the dwell time per point.

The *Ab_correction_* is given by:

(3) $$Ab_{correction}=\frac{-ln\;T}{1-T}$$

with:

(4) $$T=\frac{I_{sample+irradiator(\mathrm{cps})}-I_{sample(\mathrm{cps})}}{I_{0(\mathrm{cps})}}$$

where *I_sample_*_+_*_irradiator_* is the Zn XRF net intensity from the sample plus irradiator (cps), whereas *I_0_* is the Zn XRF net intensity from the irradiator (cps), and *I_sample_* is the Zn XRF net intensity emitted by the sample (cps). The factor T expresses the sample transmittance to Zn Kα radiation.

The instrumental sensitivity for Zn was calculated measuring a ZnTe standard thin film manufactured by Micromatter^TM^, Canada (Serial Number 6330). The equation (5) shows how the instrumental sensitivity was calculated.

(5) $$S\;(\mathrm{cps}\;{\mathrm{\mu g}}^{-1}\;{\mathrm{cm}}^{2})=\frac{I(\mathrm{cps})}{C(\mathrm{\mu g}\;{\mathrm{cm}}^{-2})}$$

where, *I* is the Zn XRF net intensity (cps) and *C* is the Zn concentration (16.2 μg Zn cm^-2^) of the ZnTe standard thin film.

**FIGURE 3:**
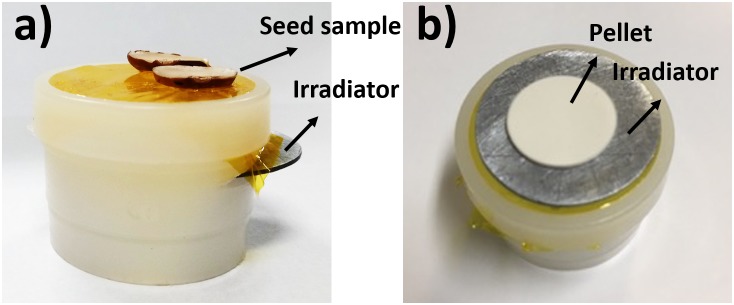
**(a)** Sample holder designed for the quantitative mapping through the emission-transmission method. The Zn disk irradiator can be easily removed and inserted for different conditions analysis. **(b)** Sample holder plus Zn disk irradiator and 200 mg Zn kg^-1^ cellulose pressed pellet. This was the experimental setup used to check the trueness of the method.

The sample holder used for this emission-transmission analysis is shown in Figure 3a. It is regular XRF cuvette modified to allow inserting and removing the Zn disk irradiator just below the sample. This is important since *I_sample_, I_sample_*_+_*_irradiator_* and *I_o_* must be measured at a fixed distance from the X-ray source and detector.

The trueness of this method was checked measuring a set of five standards: 200, 400, 600, 800, and 1000 mg Zn kg^-1^ cellulose pressed pellet. This reference pellet was prepared using cellulose binder for XRF with particle size ≤20 microns PA (SPEX, United States) spiked with 1,000 mg of Zn L^-1^ (SpecSol, Brazil) standard solution. The pellet was made transferring 150 mg of the spiked cellulose to 15 mm diameter set die and pressed at 8 ton cm^-2^ for 3 min (Spex model 3625B X-Press). The assembling of the pellet on the top of the sample holder is shown in Figure 3b.

### *In vivo* Characterization of P, S, K, and Ca Distribution in Fungi Injured Soybean Leaves

Leaves of a soybean plant were infected with spores of *Colletotrichum truncatum*. An agar plate containing the fungus was rinsed with deionized water to suspend the spores, then this suspension was filtered. Finally, the spores were spread on the adaxial face of the leaves using a Drigalski spatula.

The leaves were moistened by water spraying and the plants were incubated in plastic bags for 3 days. This procedure aimed at maintaining constant and adequate humidity for the inoculated fungus. Then, the plants were assembled in a homemade acrylic sample holder that was specially designed to keep the plant alive, in which the leaves were stretched for the analysis (see Supplementary Figure 4). The symptoms of the infection on leaves were daily monitored.

A region of 2 × 1.54 mm^2^, which comprised the fungi caused injury, was selected. The spatial distribution of the elements P, S, K, and Ca was *in vivo* determined using μ-XRF (see Table 2 for details on the instrumental parameters). The maps were recorded on the 3rd, 4th, and 5th day after the pathogen exposure.

### *In vivo* Root Uptake and Transport of Fe and Mn

*Phaseolus vulgaris* plants were cultivated using vermiculite in a growth room at 27°C and photoperiod of 12 h under LED lamps illumination of 6500 K, which supplied 250 μmol photons m^-2^ s^-1^. At V3 stage, their roots were immersed in a solution containing both monohydrated FeSO_4_ (9 × 10^-4^ M) and MnSO_4_ (9 × 10^-4^ M).

**FIGURE 4:**
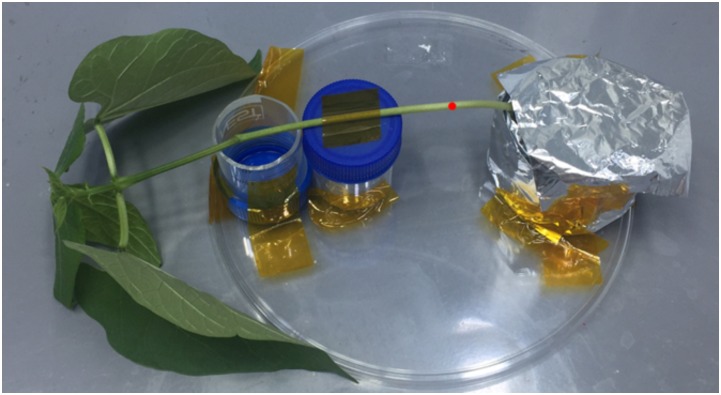
Sample holder used to load the plant inside the benchtop μ-XRF equipment, the red point represents the 1 mm spot analysis.

The plants were assembled in a homemade sample holder. The flask containing the solution was covered with aluminum layers to avoid possible XRF fluorescence coming from the solution (see Figure 4). The measurements were carried out during 48 h of constant root exposure. After each measurement, the plants were returned to the growth chamber. The measurements were performed at the stem, approximately 25 mm above of the root crown. Two plants were examined, and no signs of radiation induced damage were observed during the analysis.

### μ-XRF Showing the Spatial Distribution of Pb in *Eucalyptus* Hybrid Leaf Cultivated *in vitro*

Seeds of *Eucalyptus urophylla* × *E. grandis* hybrid were germinated and cultivated *in vitro* in a solid JADS culture media (Correia et al., 1995) for 50 days in a growth room. Roots were removed, and the shoots were transferred to the same culture medium supplemented with 0.5 g of 6BAP, 0.6% agar, 3% sucrose with the pH adjusted to 5.8. Every 21 days the plant material was transferred to a new bottle for nutritional supply. After four subcultures, the explants were transferred to a new culture media supplemented with 1,000 mg Pb (NO_3_)_2_ L^-1^ remaining exposed to this salt for 7 days.

A leaf from middle region of the shoot was cut from the plant and placed on a sample holder (see Supplementary Figures 5A,B). The μ-XRF measurement was performed using a fresh leaf. The mean background (BG_mean_, cps) under the corresponding Pb Lα peak was determined through ten randomly points selected in the leaf mapped region. The analytical signal was separated from the background using the threshold equation (2).

The sensitivity, S (cps μg^-1^cm^2^) for Pb was calculated using a Pb thin film standard Micromatter (Serial Number 6331) containing 48.3 μg Pb cm^-2^ (like shown in equation 5). The quantification of Pb was carried out considering that the leaf was an infinitely thin sample. This assumption was verified measuring the attenuation of the Lα radiation emitted by a Pb disk (Ø 25 mm × 1 mm) by several leaf samples. The Tukey test at 95% confidence interval was performed to compare the obtained means.

The concentration of Pb in the leaves was determined using the equation (6)

(6) $$C(\mathrm{\mu g}\;g^{-1})=\left(\frac{I_{sample(\mathrm{cps})}}{D(g\;{\mathrm{cm}}^{-2})\;S(\mathrm{cps}\;{\mathrm{\mu g}}^{-1}\;{\mathrm{cm}}^{-2})}\right)$$

where I_sample_ (cps) is the XRF net intensity of Pb Lα in each point of the map and D is the leaf surface density (g cm^-2^).

The trueness of the method was evaluated by measuring a thin cellulose pellet (0.0184 g cm^-2^) spiked at 1000 mg Pb kg^-1^. Five spectra were recorded at the same instrumental conditions employed for the leaf maps.

In addition to the quantitative Pb spatial distribution maps, the chemical images for K and Ca are presented. Also, the correlation between Pb, Ca, and K was accessed through scatter plots.

## Results and Discussion

### Quantitative Zn Mapping in Primed Bean Seeds

The spatial determination of minerals present in seeds can strengthen biofortification efforts, improve the understanding of the elemental redistribution during seed processing, steps such as grain polishing and crushing. Altogether, this can ensure that nutrients will reach the final consumer (Gregory et al., 2017; Mathers et al., 2017; Zaman et al., 2018).

Synchrotron-based μ-XRF was used to investigate the localization of Zn in harvested grains of wheat that was biofortified through foliar Zn application (Ajiboye et al., 2015). μ-XRF has also been used to measure the mineral distribution of seeds during its development (Iwai et al., 2012), evaluating the elemental distribution between distinct genotypes (Singh et al., 2013), and determine the location of elements in rice grains (Kyriacou et al., 2014). Here we show the quantitative distribution of Zn in two bean seeds exposed to ZnSO_4_ using a μ-XRF laboratory benchtop facility and the emission-transmission quantitative method.

The emission-transmission method requires the determination of three independent XRF net intensities: sample (cps), irradiator (cps), and sample+irradiator (cps). In the present study, two maps were enough to yield these three values, since in the map for the sample+irradiator condition part of the mapped area was not covered by the seed, and thus it gave the irradiation intensity (I_0_).

**FIGURE 5:**
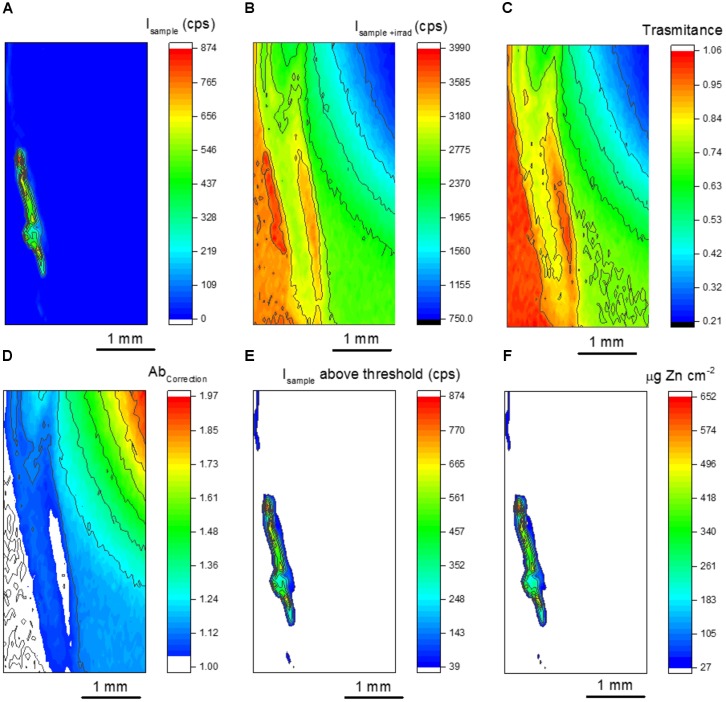
Chemical images from the data processing to obtain quantitative seed maps. **(A)** μ-XRF map for the primed seed, **(B)** μ-XRF map for the primed seed on the top the Zn disk irradiator, **(C)** calculated sample transmittance, **(D)** calculated sample absorption correction factor, **(E)** μ-XRF map for the primed seed displaying the intensity values above the quantitation counting threshold and **(F)** quantitative map showing the spatial distribution of Zn.

Figure 5 presents the steps involved in data processing. Figures 5A,B show the XRF maps recorded for I_sample_ and I_sample+irradiator_, respectively. By applying equation 4 to each pixel of these maps, one obtains the sample transmittance shown in Figure 5C. The regions of transmittance equal to 1 corresponds to the part of the irradiator not covered by the seed sample. Ideally, transmittance values above 1 should not be observed. Considering the 30 μm beam size, we believe that it was caused by micro heterogeneities of the irradiator. Another alternative to correct it consists in increasing the dwell time per pixel, which would improve the precision of the XRF counts.

Figure 5D resulted from equation 3, it is the inverse of the well-known absorption factor found the fundamental equation of XRF. Figures 5C,D shows that despite the slicing, the sample thickness was not homogenous. Figure 5E presents Zn Kα XRF intensity above the quantitation threshold. These values were obtained by applying equation 2 in Figure 5A. Finally, Figure 5F results from applying equation 1 to Figures 5D,E.

**FIGURE 6:**
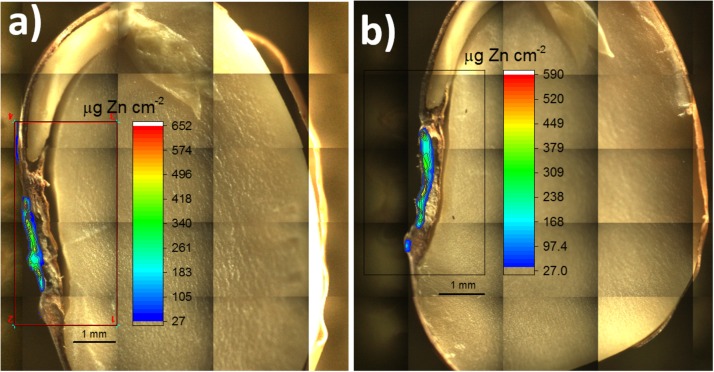
Mapped area of two primed common bean seeds. The rectangles comprises the chemical images showing the quantitative 2D distribution of Zn incorporated via seed priming with ZnSO_4(aq)_. In **(a)** is presented a sliced seed sample and **(b)** shows a hemi seed. The images show that Zn remained mostly trapped in the seed coat.

Figure 6 shows the mapped area (rectangle) for two primed bean seeds overlaid by the corresponding quantitative Zn chemical images. The seed shown in Figure 6a was sliced intending to avoid any spectral artifact coming from the seed back, whereas Figure 6b shows the image for a hemi-seed. Despite observing no spectral artifacts when analyzing a whole seed, the slicing procedure is still recommended. Since the X-ray escape depth increases as function of energy, the probability of having X-rays coming from the seed back would increase for a same line series (K or L, for instance) as function of the atomic number. This, in its turn, could lead to misinterpretation of the data, because the X-rays coming from the outer seed coat would yield a chemical image suggesting that the priming nutrient crossed the seed coat and penetrating in the endosperm. This process is illustrated in Supplementary Figure 2. We previously observed this type or artifact for molybdenum (Kα 17,480 eV) soybean treated seeds (not shown here).

The trueness of this method was evaluated using a set of five standards (Supplementary Figure 6). The recovery ranged from 91 to 106%. Considering the pellet measurement, the limit of quantification (10σ) of the method was 1.6 μg Zn cm^-2^.

Most of Zn remained trapped in the seed coat, even though the seed priming solution delivered a soluble Zn form. It was observed a Zn hotspot (reddish area) in the hilum region (spongy tissue that allows water uptake into the seed).

Measurements of intermediate thickness samples using the emission-transmission method can be interesting for environmental (Markowicz et al., 1996), geological (Funtua, 1999), biological (Funtua, 1999; Bamford et al., 2004; Bako et al., 2008), and drug (Mahawatte et al., 2006) samples. In plant science, this method was applied to quantify the elements in healthy and fumagine infected orange and lemon leaves. The quantification of Ti, Mn, Fe, Cu, and Zn gave limits of detection that ranged from 1 to 10 μg g^-1^ (Blonski et al., 2006). The same method was also employed to quantify inorganic elements in tobacco leaves (Oyewale et al., 2002) and radish plants (Gupta et al., 2007).

Regarding alternative techniques, simultaneous particle-induced X-ray emission using a focused ion beam (μ-PIXE), Rutherford backscattering (RBS) and scanning transmission ion microscopy (STIM) analyses yielded quantitative map of metal concentration in *Arabidopsis thaliana* seeds (Schnell Ramos et al., 2013).

### *In vivo* Characterization of P, S, K, and Ca Distribution in Fungi Injured Soybean Leaves

X-ray fluorescence microprobe can support understanding of chemical composition of the infected tissues by bacteria, fungi, virus, and their proliferation in plants while alive. It support the understanding which elements plants may redistribute to oppose the invasion, or the nutrients that pathogens take up to develop. This information enables to find management strategies which mimics the self-defense mechanism of plants. In principle, following such spatial distribution patterns, one could recognize infections even before the appearance of visual symptoms and therefore avoid losses.

**FIGURE 7:**
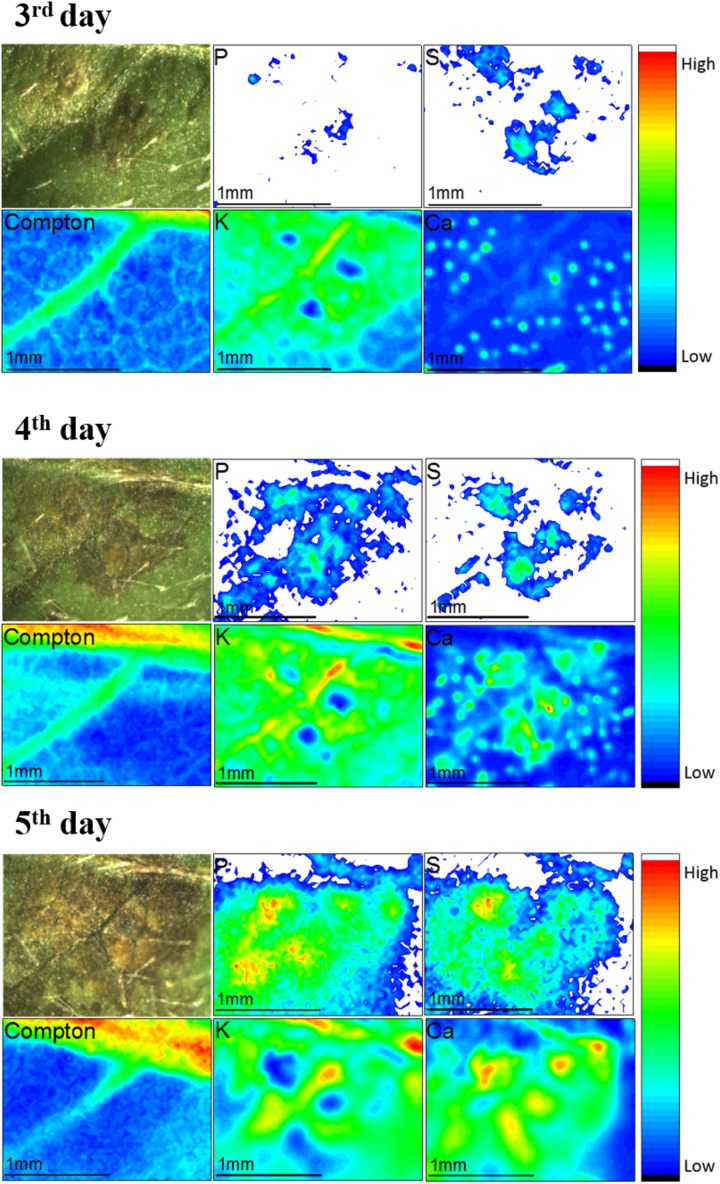
μ-XRF monitoring the evolution of *Colletotrichum truncatum* on the surface of the soybean from the 3^rd^ to the 5^th^ day after the fungi inoculation. The images show pictures and chemical images for P, S, Compton scattering, K and Ca. The nutrient distribution pattern changes as the disease spreads. The unit of the scale the right side of the maps is counts per second (cps).

Figure 7 shows disease development from the 3rd to the 5th day of fungi infection. The images present a picture of the soybean leaf, the infected region corresponds to the darker areas. The other figures are chemical images that unravel the spatial distribution of P, S, K, and Ca in the same of area shown by the picture. Additionally, we also show the Rh Kα Compton scattering map that indicate the presence of veins in the leaf. The dynamic chemical images showed that P, S, and Ca trended to accumulate in the injured area as the disease spreads. On the contrary, K seems to be depleted in the region attacked by the fungi.

Although there are few studies reporting the spatial distribution of elements during diseases, this behavior is probably correlated to a defense mechanism of the plant (Taiz and Zeiger, 2013). The optical image demonstrates the existence of a pre-damaged area around the pathogen where the calcium concentration was higher. Since the fungi is a necrotrophic pathogen (Ranathunge et al., 2012), the defense response of the plant consists in isolating the infected area avoiding the spread of the disease to the rest of the plant in tissue (Taiz and Zeiger, 2013). Nürnberger et al. (1994) showed that elicitors (molecule extrinsic coming from pathogens connect with plant proteins triggering defense mechanism) induces the influx of Ca^2+^ and efflux of K^+^ to cells (Nürnberger et al., 1994). Other study showed that Ca^2+^ triggers the cell death program (Atkinson et al., 1990; Tavernier et al., 1995; Lamb and Dixon, 1997), then the increase of Ca XRF intensity might be linked to this mechanism.

### *In vivo* Root Uptake and Transport of Fe and Mn

In this section, we show an example of how μ-XRF can be used to *in vivo* trace the absorption and transport of Mn and Fe in *P. vulgaris*. Although synchrotron XRF was previously used to monitor the uptake of Ni, Mn, and Cr (Hwang et al., 2016) and thallium (Scheckel et al., 2004), to the best of our knowledge, this is the first report monitoring the simultaneous uptake of two nutrients in a living plant using a benchtop XRF equipment.

Basically, three strategies are used to monitor the root to shoot uptake of elements in solution. Firstly, one can monitor the absorption using radiolabeled elements (von Wiren et al., 1995; Hart et al., 1998; Rengel et al., 1998). Additionally, it is possible to monitor the depletion of the target element in the solution to which roots are exposed (Hart et al., 1998). Finally, one can also collect the vegetal tissues from time to time and determine the concentration of the desired elements trough chemical or instrumental analysis (Avenue and Africa, 1973; Puccinelli et al., 2017).

Radioisotopes grant researchers the possibility of monitoring the uptake and transport of nutrients while the plant is alive, however, their manipulation involves many issues regarding safety and are not easy to acquire. The collection of aliquots from the solution in which roots are exposed allows tracing whether nutrients were absorbed, however, this procedure does not allow inferring about transport. Finally, the analysis of the tissues collected from several different individuals can introduce errors to the experiment due to intrinsic differences that they may present, moreover this procedure involves a large number of samples, it is also laborious and time consuming since several steps are involved for sample preparation and analysis.

In this context, μ-XRF can be useful to investigate the mechanisms of nutrient uptake and transport. The elements can be simultaneously monitored, and the X-ray beam can probe different parts of roots, stem, or leaves. Last but not least, the measurements can be carried out *in vivo* plants. The continuously increasing uptake tendency for Mn and Fe, in addition to previously published studies suggested that no measurable radiation damaged occurred (da Cruz et al., 2017; Gomes et al., 2017).

**FIGURE 8:**
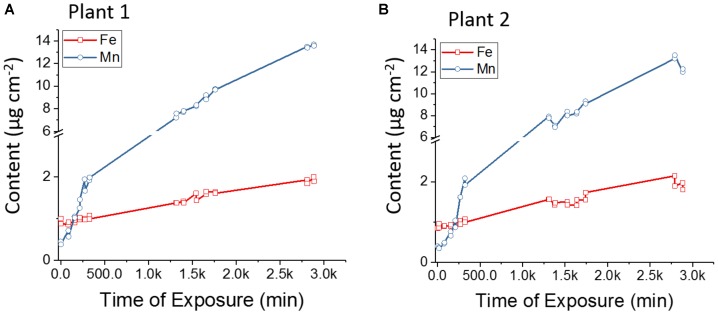
**(A)** and **(B)** Biological replicates of *in vivo* X-ray fluorescence monitoring the competitive absorption and translocation of Fe and Mn in the stem of *Phaseolus vulgaris*. The number of counts was normalized by the elemental sensitivity which allows comparing their contents quantitatively. Plant roots were immersed in aqueous solution of FeSO_4_ and MnSO_4_ (both at 9 × 10^-4^ mol L^-1^). The results show that Mn uptake and transport is faster than Fe.

In the present study the absorption and transport rates of Fe and Mn were monitored *in vivo*. Figure 8 shows the number of counts for Fe-Kα and Mn-Kα, normalized by the sensitivity of each element, as a function of exposure time (Figures 8A,B are biological replicate). The plants showed an initial content of Fe greater than Mn, but approximately after 250 min of exposure, Mn was higher than Fe. It was also observed during the 48 h that the increase of Mn content was much faster than for Fe, showing a preference of the plants for Mn uptake.

The observed results might be explained by the competitive inhibition reported in the literature (Malavolta et al., 1997; Alam et al., 2000), where Fe and Mn compete for the same transport sites due to their physical and chemical similarities, such as atomic masses, ionic radius and electronic structure (Madejczyk and Ballatori, 2012). IRT1 is a transporter protein member of the ZIP family, it is known as Fe^2+^ transporter, however, assays carried out in yeast (Korshunova et al., 1999) and barley (Pedas et al., 2008) showed that this protein can also transport Mn by electrochemical active influx (Korshunova et al., 1999). Likewise, YSL is responsible to Fe uptake (Curie et al., 2001), but also translocates Mn complexed with nicotianamine (Andresen et al., 2018). Additionally, it was verified in rice that the transporter protein NRAMP5 also contributes to Mn and Fe transport, being another entry route for these micronutrients (Ishimaru et al., 2012).

Table 3 presents the Fe and Mn absorption velocity expressed by counts min^-1^. Therefore, it is possible to verify that the increasing content of Fe and Mn in the stem followed a linear function of the time. The slopes showed that absorption and transport of Mn was nearly 10-fold faster than for Fe. Manganese transport can occur by transporters or pumps, and these two mechanisms may coexist, while Fe uptake in beans only takes place trough transporters after Fe^2+^ reduction (Andresen et al., 2018). The 9 × 10^-4^ mol L^-1^ concentration is higher than commonly used in Hoagland’s solution (Hoagland and Arnon, 1950), and the literature has shown that Mn toxicity decreased the translocation of absorbed Fe to shoots (Alam et al., 2000), and also the elevated availability of Mn induces Fe deficiency (Eroglu et al., 2016).

**Table 3:** Fe and Mn absorption velocity by *Phaseolus vulgaris* in solution containing FeSO_4_ (9 × 10^-4^ M^1^) and MnSO_4_ (9 × 10^-4^ M^-1^).

Plant	Element	Slope × 10^-3^ (counts min^-1^)	*R*^2^
1	Fe	1.27 ± 0.06	0.95
	Mn	12.1 ± 0.4	0.98
2	Fe	1.21 ± 0.04	0.97
	Mn	12.9 ± 0.1	0.99


### μ-XRF as Direct Determination of Pb in *Eucalyptus* Hybrid Leaf Cultivated *in vitro*

There is much expectation that fast growing plants might be used for bioremediation of soils (Couselo et al., 2012). Due to its high biomass rate production and stable organic tissue, these species may act as sinks extracting potentially toxic element from the soil. Several species were evaluated in phytoremediation studies, mostly from temperate climate as *Populus* (Di Lonardo et al., 2011), *Brassica* (Nehnevajova et al., 2007), and *Salix* (Lyyra et al., 2006) genus.

The screening and selection of genotypes with potential for bioremediation can be carried out through *in vitro* studies (Nehnevajova et al., 2007; Doran, 2009; Di Lonardo et al., 2011). The determination of the spatial distribution of elements can show possible synergism or antagonism between the potentially toxic elements that one intends to remove from soil and the mineral nutrients in the plant (Bueno Guerra et al., 2013). Imaging techniques can be used to evaluate such interactions (Campos et al., 2015). The main techniques currently used are LA-ICP-MS (Tian et al., 2011), μ-XRF (Campos et al., 2015), synchrotron XRF (Hokura et al., 2006), and XRF microtomography (Terzano et al., 2008).

**FIGURE 9:**
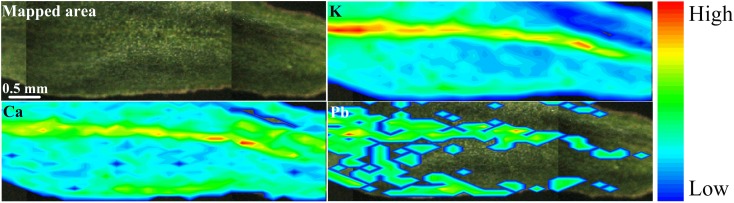
μ-XRF chemical images showing the spatial distribution of K, Ca, and Pb in the leaf of *Eucalyptus urophylla* × *E. grandis*. Potassium and Ca were found in the whole tissue, although more concentrated in the midrib. On the other hand, Pb accumulated in the midrib and in some hotspots along the leaf border. Scale bar: 0.5 mm.

Figure 9 shows that after 7 days of exposure to Pb(NO_3_)_2_, Pb was absorbed and stored in the leaves. Fast absorption and translocation to the upper parts are important for the phytoremediation process since it reduces the bioremediation time (Nedelkoska and Doran, 2000). The highest intensities for K, Ca and Pb were found mainly at the midrib and leaf margins.

**FIGURE 10:**
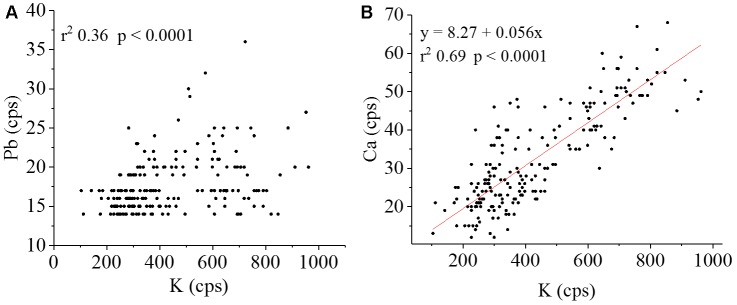
Pearson’s correlation (position vs. intensity) scatter plots for **(A)** Pb Lα vs. K Kα and **(B)** the regression equation K Kα vs. Ca Kα Lα (number of points = 208; α = 0.01). K and Ca show high spatial correlation, this was not observed for K and Pb.

Figure 10A indicates a weak positive correlation between Pb and K. Similar results with a higher correlation between Pb and S was founded in an accumulating ecotype (Tian et al., 2010). The Pb was also detected mostly within the vascular tissue, which suggested low mobility out of this region. This highlights the importance of K for Pb transport and toxicity control, in which the macronutrient is one of the mostly important element for osmotic cell wall and vacuole storage (Marschner, 1995). On the other hand, a strong positive correlation was found between K and Ca (Figure 10B).

The XRF absorption factor was assessed for Pb in the *Eucalyptus* leaves. Table 4 presents the intensity of Pb Lα XRF peak for the irradiator (Pb disk) and irradiator covered by a leaf. The leaf blade and midrib attenuated 0.08 and 0.55% of the photons emitted by the disk, respectively. In most cases, attenuation values up to 10% can be neglected, since in this condition the concentration of the analyte and the XRF signal hold a linear relationship. Therefore, in the present situation the *Eucalyptus* leaf can be considered an infinitely thin sample, it means that the absorption correction (Ab_correction_) factor is equal to one and the Pb concentration can determined directly from its XRF intensity and sensitivity, as shown in Equation 1.

**Table 4:** Infinitely thin film thickness test (α = 0.05) for Pb in *Eucalyptus* hybrid leaf.

Analyzed region	Pb net XRF counts
Irradiator	2,557^a^
Irradiator + leaf blade	2,555^a^
Irradiator + leaf midribs	2,543^a^


*Five replicates were recorded. ^∗^Values followed by the same letter do not present statistical difference under Tukey test at 95% confidence interval.*

**FIGURE 11:**
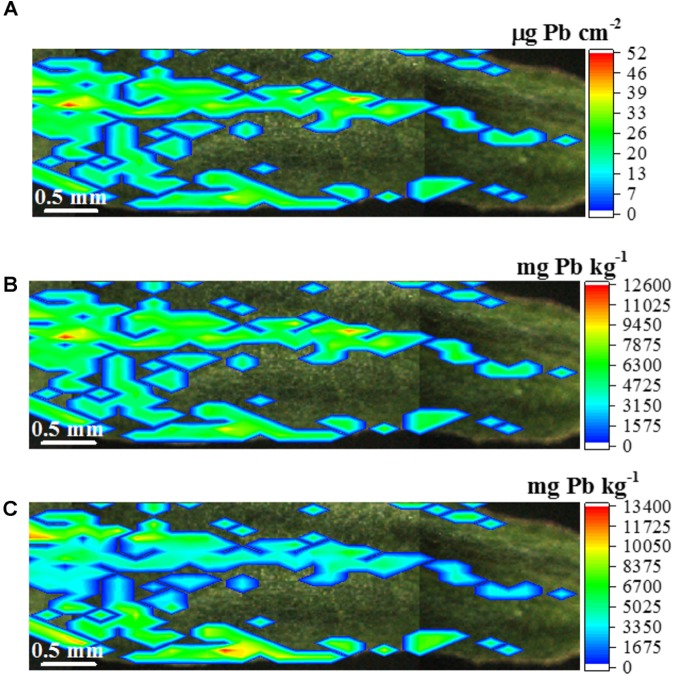
μ-XRF quantitative map displaying the concentration of Pb in the leaf of *Eucalyptus urophylla* × *E. grandis*. Lead is not homogenously accumulated in the leaf tissue, panel **(A)** presents the Pb concentration in weight/area unity (mg Pb cm^-2^ leaf surface), panel **(B)** shows the Pb map in weight/weight (mg Pb kg^-1^ fresh tissue) unity considering a homogenous leaf surface density, and panel **(C)** shows the corrected map taking into account the difference between the thickness the leaf blade and midrib. Scale bar: 0.5 mm.

Figure 11 shows that regardless the approach chosen to transform the Pb Lα signal in concentration, one can clearly observe that the Pb storage is not homogenous along the leaf tissues. The accumulation was found mainly at the midrib and leaf margins, following the petiole direction toward the leaf tip.

Figure 11A expresses the Pb concentration in weight/area (μg cm^-2^) unity such as in Figure 6. Figure 11B presents an attempt of transforming the weigh/area concentration in a more common weight/weight concentration unity (mg kg^-1^). This step was accomplished by dividing the weight/area concentration by the average leaf surface density (g cm^-2^). However, Supplementary Figure 7 and Table 4 show that the midrib is nearly two-fold thicker than the leaf blade. This fact makes the assumption of a homogenous surface density only an approximation. Aiming at accounting for this factor, Figure 11C presents the Pb concentration along the leaf sample considering the specific surface densities for midrib and leaf blade.

Figure 11C shows that the Pb concentration varies from 4,880 to 13,400 mg Pb kg^-1^ fresh tissue weight. The median concentration of the Pb spots was 5,586 mg Pb kg^-1^. These high concentrations values might be explained by the removal of the plant radicle. The stem was directly in contact with the spiked culture medium, which might have increased the phytoextraction potential. Anyway, it is curious to note that after 7 days of Pb exposure, the explants did not present any noticeable symptom of phytotoxicity. The recovery of the quantitative method was 108%.

The literature shows that *Brassica pekinensis*, a Pb-hyperaccumulator species, exposed to 500 mg Pb kg^-1^ in a culture media for 2 weeks accumulated in average 1,220 mg Pb kg^-1^ dry weight at the leaves (Xiong, 1998). Other studies showed Pb leaves content of 95.92, 75.37, 136.67, 304.39, and 89.84 mg kg^-1^ dry weight for *Salix cathayana, Carpinus wangii, Lithocarpus dealbatus, Llex plyneura*, and *Sambucus chinensis*, respectively (Yanqun et al., 2004).

One has to keep in mind that the concentration values supplied by analytical techniques such as atomic emission and absorption spectroscopies regards the average concentration of the element diluted in the whole sample tissue. It is likely that in these above-mentioned studies, Pb was not evenly distributed in leaves, but forming hotspots such as shown in the present paper.

This heterogeneous distribution in the leaf blade may suggest the presence tissue compartmentation of Pb. These maps can help to explain the mechanisms of tolerance presented by the *Eucalyptus* shoots (Nedelkoska and Doran, 2000), an important feature for maintaining growth, for the detoxification, and bioremediation process (Hall, 2002; Couselo et al., 2012).

## Conclusion

Benchtop μ-XRF is a versatile tool in plant science. It can be used to monitor the mineral elemental composition of a single point or produce lines and 2D images. As an elemental imaging tool, it is complementary to SEM-EDS and TEM-EDS, since it covers a larger sample area, from hundreds of μm^2^ to cm^2^, and presents better limits of detection for most elements.

As a unique feature, μ-XRF allows for non-destructive analysis of fresh and living plant tissues bringing the possibility of dynamic and *in vivo* studies. Monitoring nutrient uptake kinetics while the plant is alive is possible using the technology described here. In biofortification or seed treatment effort, this tool allows to evaluate the spatial distribution of different elements and thus verifying the trueness of both, biofortification and seed treatments. In pathogen study, the chemical image allowed to verify changes in chemical distribution of elements such as K, P, S, and Ca in leaves infected. Bioremediation effort this technique allows to verify where is allocate the element and what concentration in plant.

## Author Contributions

ER organized and wrote most of the manuscript. ND and JC carried out the emission transmission quantitative analysis of Zn in primed seeds. MG and ER performed the *in vivo* characterization of P, S, K, and Ca distribution in injured soybean leaves. TdC monitored the root uptake and transport of Fe and Mn. ASN determined the content of Pb in the leaf of *Eucalyptus* hybrid. SS and EdA assisted all the experiments. HC designed the study, coordinated the work, supervised the students, and reviewed the whole manuscript.

## Conflict of Interest Statement

The authors declare that the research was conducted in the absence of any commercial or financial relationships that could be construed as a potential conflict of interest.

## References

[B1] Ajiboye B. Cakmak I. Paterson D. De Jonge M. D. Howard D. L. Stacey S. P. (2015). X-ray fluorescence microscopy of zinc localization in wheat grains biofortified through foliar zinc applications at different growth stages under field conditions. *Plant Soil* 392 357–370. 10.1007/s11104-015-2467-8

[B2] Alam S. Kamei S. Kawai S. (2000). Phytosiderophore release from manganese-induced iron deficiency in barley. *J. Plant Nutr.* 23 1193–1207. 10.1080/01904160009382092

[B3] Andresen E. Peiter E. Kupper H. (2018). Trace metal metabolism in plants. *J. Exp. Bot.* 69 909–954. 10.1093/jxb/erx465 29447378

[B4] Atkinson M. M. Keppler L. D. Orlandi E. W. Baker C. J. Mischke C. F. (1990). Involvement of plasma membrane calcium influx in bacterial induction of the K + /H + and hypersensitive responses in tobacco. *Plant Physiol.* 92 215–221. 10.1104/pp.92.1.215 16667249 PMC1062272

[B5] Avenue J. S. Africa S. (1973). Studies on mineral ion absorption by plants. *Plant Soil* 38 509–523. 10.1007/BF00010691

[B6] Bako S. Samson A. Funtua I. I. (2008). Spatial distribution and heavy metal content of some bryophytes and lichens in relation to air pollution in Nigeria’s Guinea Savanna. *Int. J. Environ. Pollut* 33 195–206. 10.1504/IJEP.2008.019393

[B7] Bamford S. A. Jaksic M. Medunic Z. Wegrzynek D. Chinea-Cano E. Markowicz A. (2004). Extending the quantitative analytical capabilities of the EDXRF technique for plant-based samples. *Xray Spectrom.* 33 277–280. 10.1002/xrs.721

[B8] Beckhoff B. Kanngießer B. Langhoff N. Wedell R. Wolff H. (eds) (2006). *Handbook of Practical X-Ray Fluorescence Analysis.* Berlin: Springer, 10.1007/978-3-540-36722-2

[B9] Beckhoff B. Kanngießer B. Langhoff N. Wedell R. Wolff H. (2007). *Handbook of Practical X-Ray Fluorescence Analysis.* Berlin: Springer.

[B10] Blonski M. S. Appoloni C. R. Parreira P. S. Arag?o P. H. A. Nascimento Filho V. F. (2006). Analysis of the chemical elements in leaves infected by fumagina by X-ray fluorescence technique. *J. Radioanal. Nucl. Chem.* 270 197–201. 10.1007/s10967-006-0340-1

[B11] Blonski M. S. Appoloni C. R. Parreira P. S. Aragão P. H. A. Filho V. F. N. (2007). Elementary chemical analysis in leaves infected by fumagina by x-ray fluorescence technique. *Braz. Arch. Biol. Technol.* 50 851–860. 10.1590/S1516-89132007000500013

[B12] Bueno Guerra M. B. Schaefer C. E. G. R. de Carvalho G. G. A. de Souza P. F. Júnior D. S. Nunes L. C. (2013). Evaluation of micro-energy dispersive X-ray fluorescence spectrometry for the analysis of plant materials. *J. Anal. At. Spectrom.* 28 1096–1101. 10.1039/c3ja50084e

[B13] Campos N. V. Bueno Guerra M. B. Mello J. W. V. Schaefer C. E. G. R. Krug F. J. Alves E. E. N. (2015). Accumulation and spatial distribution of arsenic and phosphorus in the fern *Pityrogramma calomelanos* evaluated by micro X-ray fluorescence spectrometry. *J. Anal. At. Spectrom.* 30 2375–2383. 10.1039/C5JA00348B

[B14] Castillo-Michel H. A. Larue C. Pradas del Real A. E. Cotte M. Sarret G. (2017). Practical review on the use of synchrotron based micro- and nano- X-ray fluorescence mapping and X-ray absorption spectroscopy to investigate the interactions between plants and engineered nanomaterials. *Plant Physiol. Biochem.* 110 13–32. 10.1016/j.plaphy.2016.07.018 27475903

[B15] Correia D. Gonçalves A. N. Couto H. Y. Z. Ribeiro M. C. (1995). Efeito do meio de cultura líquido e sólido no crescimento e desenvolvimento de gemas de Eucalyptus grandis x Eucalyptus urophylla na multiplicação in vitro. *IPEF Piracicaba* 48 107–116.

[B16] Couselo J. L. Corredoira E. Vieitez A. M. Ballester A. (2012). “Plant tissue culture of fast-growing trees for phytoremediation research,” in *Plant Cell Culture Protocols. Methods in Molecular Biology (Methods and Protocols)* Vol. 877 eds Loyola-Vargas V. Ochoa-Alejo N. (Totowa, NJ: Humana Press), 247–263. 10.1007/978-1-61779-818-4_19 22610633

[B17] Curie C. Panaviene Z. Loulergue C. Dellaporta S. L. Briat J. F. Walker E. L. (2001). Maize yellow stripe1 encodes a membrane protein directly involved in Fe(III) uptake. *Nature* 409 346–349. 10.1038/35053080 11201743

[B18] da Cruz T. N. M. Savassa S. M. Gomes M. H. F. dos Santos E. Duran N. M. Almeida E. (2017). In vivo X-ray spectroscopy shedding light on the mechanisms of absorption and transport of ZnO nanoparticles by plants. *Environ. Sci. Nano* 4 2367–2376. 10.1039/C7EN00785J

[B19] Di Lonardo S. Capuana M. Arnetoli M. Gabbrielli R. Gonnelli C. (2011). Exploring the metal phytoremediation potential of three *Populus alba* L. clones using an in vitro screening. *Environ. Sci. Pollut. Res.* 18 82–90. 10.1007/s11356-010-0354-7 20563887

[B20] Doran P. M. (2009). Application of plant tissue cultures in phytoremediation research: Incentives and limitations. *Biotechnol. Bioeng.* 103 60–76. 10.1002/bit.22280 19309741

[B21] Duran N. M. Savassa S. M. Lima R. G. de Almeida E. Linhares F. S. van Gestel C. A. M. (2017). X-ray spectroscopy uncovering the effects of Cu based nanoparticle concentration and structure on *Phaseolus vulgaris* germination and seedling development. *J. Agric. Food Chem.* 65 7874–7884. 10.1021/acs.jafc.7b03014 28817280

[B22] Eroglu S. Meier B. von Wirén N. Peiter E. (2016). The vacuolar manganese transporter MTP8 determines tolerance to Iron deficiency-induced chlorosis in Arabidopsis. *Plant Physiol.* 170 1030–1045. 10.1104/pp.15.01194 26668333 PMC4734556

[B23] Fittschen U. E. A. Kunz H.-H. Höhner R. Tyssebotn I. M. B. Fittschen A. (2017). A new micro X-ray fluorescence spectrometer for *in vivo* elemental analysis in plants. *Xray Spectrom.* 46 374–381. 10.1002/xrs.2783

[B24] Funtua I. I. (1999). Application of the transmission-emission method in EDXRF for the determination of trace elements in geological and biological materials. *J. Trace Microprobe Tech.* 17 293–297.

[B25] Gomes M. H. F. Duran N. M. Cassanji J. G. B. Rodrigues E. S. da Cruz T. N. M. Almeida E. (2017). In vivo XRF probing the absorption and translocation of nutrients in seeds and plants. *EDAX Insight* 15 1–3.

[B26] Gregory P. J. Wahbi A. Adu-Gyamfi J. Heiling M. Gruber R. Joy E. J. M. (2017). Approaches to reduce zinc and iron deficits in food systems. *Glob. Food Sec.* 15 1–10. 10.1016/j.gfs.2017.03.003

[B27] Guerra M. Adame A. de Almeida E. Brasil M. Schaefer C. Krug F. (2017). In situ determination of K, Ca, S and Si in fresh sugar cane leaves by handheld energy dispersive X-Ray fluorescence spectrometry. *J. Braz. Chem. Soc.* 29 1–27. 10.21577/0103-5053.20170229

[B28] Gupta D. Chatterjee J. M. Ghosh R. Mitra A. K. Roy S. Sarkar M. (2007). Elemental uptake of radish grown near a Municipal Solid Waste dumping site by EDXRF *J. Radioanal. Nucl. Chem* 274 389–395. 10.1007/s10967-007-1127-8

[B29] Hall J. L. (2002). Cellular mechanisms for heavy metal detoxification and tolerance. *J. Exp. Bot.* 53 1–11. 10.1093/jxb/53.366.1 11741035

[B30] Hart J. J. Welch R. M. Norvell W. A. Sullivan L. A. Kochian L. V. (1998). Characterization of cadmium binding, uptake, and translocation in intact seedlings of bread and durum wheat cultivars. *Plant Physiol.* 116 1413–1420. 10.1104/pp.116.4.1413 9536059 PMC35049

[B31] Haschke M. (2014). *Laboratory Micro-X-Ray Fluorescence Spectroscopy.* Cham: Springer International Publishing, 10.1007/978-3-319-04864-2

[B32] Henderson R. (1990). Cryo-protection of protein crystals against radiation damage in electron and X-ray diffraction. *Proc. R. Soc. Lond. B* 241 6–8. 10.1098/rspb.1990.0057

[B33] Hoagland D. R. Arnon D. I. (1950). *The Water-Culture Method for Growing Plants Without Soil.* Berkeley, CA: University of California.

[B34] Hokura A. Omuma R. Terada Y. Kitajima N. Abe T. Saito H. (2006). Arsenic distribution and speciation in an arsenic hyperaccumulator fern by X-ray spectrometry utilizing a synchrotron radiation source. *J. Anal. At. Spectrom.* 21 321–328. 10.1039/b512792k

[B35] Hwang B. G. Lee S. J. Gil K. (2016). In-vivo analysis of the uptake process of heavy metals through maize roots by using synchrotron X-ray fluorescence spectroscopy. *J. Korean Phys. Soc.* 69 1824–1829. 10.3938/jkps.69.1824

[B36] Ishimaru Y. Takahashi R. Bashir K. Shimo H. Senoura T. Sugimoto K. (2012). Characterizing the role of rice NRAMP5 in Manganese. Iron and Cadmium Transport. *Sci. Rep.* 2:286. 10.1038/srep00286 22368778 PMC3285952

[B37] Iwai T. Takahashi M. Oda K. Terada Y. Yoshida K. T. (2012). Dynamic changes in the distribution of minerals in relation to phytic acid accumulation during rice seed development. *Plant Physiol.* 160 2007–2014. 10.1104/pp.112.206573 23090587 PMC3510127

[B38] Janssens K. H. A. Adams F. Rindby A. (2000). *Microscopic x-ray Fluorescence Analysis.* Hoboken, NJ: John Wiley & Sons.

[B39] Kalcsits L. A. (2016). Non-destructive measurement of calcium and potassium in apple and pear using handheld X-ray fluorescence. *Front. Plant Sci.* 7:442. 10.3389/fpls.2016.00442 27092160 PMC4820457

[B40] Korshunova Y. O. Eide D. Clark W. G. Guerinot M. L. Pakrasi H. B. (1999). The IRT1 protein from *Arabidopsis thaliana* is a metal transporter with a broad substrate range. *Plant Mol. Biol.* 40 37–44. 10.1023/A:1026438615520 10394943

[B41] Kyriacou B. Moore K. L. Paterson D. de Jonge M. D. Howard D. L. Stangoulis J. (2014). Localization of iron in rice grain using synchrotron X-ray fluorescence microscopy and high resolution secondary ion mass spectrometry. *J. Cereal Sci.* 59 173–180. 10.1016/j.jcs.2013.12.006

[B42] Lamb C. Dixon R. A. (1997). The oxidative burst in plant disease resistance. *Annu. Rev. Plant Biol.* 48 251–275. 10.1146/annurev.arplant.48.1.251 15012264

[B43] Leroux J. Mahmud M. (1966). X-ray quantitative analysis by an emission-transmission method. *Anal. Chem.* 38 76–82. 10.1021/ac60233a021 17268013

[B44] Lyyra S. Lima A. Merkle S. A. (2006). In vitro regeneration of *Salix nigra* from adventitious shoots. *Tree Physiol.* 26 969–975. 10.1093/treephys/26.7.969 16585042

[B45] Madejczyk M. S. Ballatori N. (2012). The iron transporter ferroportin can also function as a manganese exporter. *Biochim. Biophys. Acta* 1818 651–657. 10.1016/j.bbamem.2011.12.002 22178646 PMC5695046

[B46] Mahawatte P. Dissanayaka K. R. Hewamanna R. (2006). Elemental concentrations of some Ayurvedic drugs using energy dispersive XRF. *J. Radioanal. Nucl. Chem* 270 657–660. 10.1007/s10967-006-0444-7

[B47] Malavolta E. Vitti G. C. Oliveira S. A. (1997). *Avaliação do Estado Nutricional das Plantas: Princiìpios e Aplicações*, 2nd Edn. Piracicaba: POTAFOS.

[B48] Margui E. (2013). *X-Ray Fluorescence Spectrometry and Related Techniques.* New York, NY: Momentum Press, 10.5643/9781606503935

[B49] Markowicz A. Haselberger N. Dargie M. Tajani A. Tchantchane A. Valkovic V. (1996). Application of X-ray fluorescence spectrometry in assessment of environmental pollution. *J. Radioanal. Nucl. Chem. Artic.* 206 269–277. 10.1007/BF02039653

[B50] Marschner H. (1995). *Mineral Nutrition of Higher Plants*, 2nd Edn. Cambridge, MA: Academic Press.

[B51] Mathers A. W. Young S. D. McGrath S. P. Zhao F. J. Crout N. M. J. Bailey E. H. (2017). Determining the fate of selenium in wheat biofortification: an isotopically labelled field trial study. *Plant Soil* 1 1–17. 10.1007/s11104-017-3374-y

[B52] McLaren T. I. Guppy C. N. Tighe M. K. Forster N. Grave P. Lisle L. M. (2012). Rapid, nondestructive total elemental analysis of vertisol soils using portable X-ray fluorescence. *Soil Sci. Soc. Am. J.* 76 1436–1445. 10.2136/sssaj2011.0354

[B53] Navas M. J. Asuero A. G. Jiménez A. M. (2016). A review of energy dispersive X-ray fluorescence (EDXRF) as an analytical tool in numismatic studies. *Appl. Spectrosc.* 70 207–221. 10.1177/0003702815616594 26767646

[B54] Nedelkoska T. V. Doran P. M. (2000). Characteristics of heavy metal uptake by plant specie∼ with potential for phytoremediation and phytomining. *Miner. Eng.* 13 549–561. 10.1016/S0892-6875(00)00035-2 27854061

[B55] Nehnevajova E. Herzig R. Erismann K. H. Schwitzguébel J. P. (2007). In vitro breeding of *Brassica juncea* L. to enhance metal accumulation and extraction properties. *Plant Cell Rep.* 26 429–437. 10.1007/s00299-006-0264-9 17103002

[B56] Nürnberger T. Nennstiel D. Jabs T. Sacks W. R. Hahlbrock K. Scheel D. (1994). High affinity binding of a fungal oligopeptide elicitor to parsley plasma membranes triggers multiple defense responses. *Cell* 78 449–460. 10.1016/0092-8674(94)90423-5 8062387

[B57] Oyewale A. O. Funtua I. I. Ekwumemgbo P. (2002). Energy dispersive X-ray fluorescence spectrometry analysis of elements in tobacco cigarette and ashed-tobacco samples. *J. Sci. Ind. Res.* 61 48–52.

[B58] Pedas P. Ytting C. K. Fuglsang A. T. Jahn T. P. Schjoerring J. K. Husted S. (2008). Manganese efficiency in barley: identification and characterization of the metal ion transporter HvIRT1. *Plant Physiol.* 148 455–466. 10.1104/pp.108.118851 18614714 PMC2528110

[B59] Puccinelli M. Malorgio F. Rosellini I. Pezzarossa B. (2017). Uptake and partitioning of selenium in basil (*Ocimum basilicum* L.) plants grown in hydroponics. *Sci. Hortic.* 225 271–276. 10.1016/j.scienta.2017.07.014

[B60] Ranathunge N. P. Mongkolporn O. Ford R. Taylor P. W. J. (2012). *Colletotrichum truncatum* Pathosystem on Capsicum spp: infection, colonization and defence mechanisms. *Australas. Plant Pathol* 41 463–473. 10.1007/s13313-012-0156-0

[B61] Rengel Z. Römheld V. Marschner H. (1998). Uptake of zinc and iron by wheat genotypes differing in tolerance to zinc deficiency. *J. Plant Physiol.* 152 433–438. 10.1016/S0176-1617(98)80260-5

[B62] Scheckel K. G. Lombi E. Rock S. A. McLaughlin M. J. (2004). In Vivo synchrotron study of thallium speciation and compartmentation in Iberis intermedia. *Environ. Sci. Technol.* 38 5095–5100. 10.1021/es049569g 15506204

[B63] Schnell Ramos M. Khodja H. Mary V. Thomine S. (2013). Using μPIXE for quantitative mapping of metal concentration in *Arabidopsis thaliana* seeds. *Front. Plant Sci.* 4:168. 10.3389/fpls.2013.00168 23761799 PMC3669754

[B64] Singh S. P. Vogel-Mikuš K. Arčon I. Vavpetič P. Jeromel L. Pelicon P. (2013). Pattern of iron distribution in maternal and filial tissues in wheat grains with contrasting levels of iron. *J. Exp. Bot.* 64 3249–3260. 10.1093/jxb/ert160 23918965 PMC3733147

[B65] Taiz L. Zeiger E. (2013). *Fisiologia Vegetal*, 5th Edn. Porto Alegre: Artmed.

[B66] Tavernier E. Wendehenne D. Blein J.-P. Pugin A. (1995). Involvement of free calcium in action of cryptogein, a proteinaceous elicitor of hypersensitive reaction in tobacco cells. *Plant Physiol.* 109 1025–1031. 10.1104/pp.109.3.1025 12228650 PMC161405

[B67] Terzano R. Al Chami Z. Vekemans B. Janssens K. Miano T. Ruggiero P. (2008). Zinc distribution and speciation within rocket plants (*Eruca vesicaria* L. Cavalieri) grown on a polluted soil amended with compost as determined by XRF microtomography and Micro-XANES. *J. Agric. Food Chem.* 56 3222–3231. 10.1021/jf073304e 18410113

[B68] Tian S. Lu L. Labavitch J. Yang X. He Z. Hu H. (2011). Cellular sequestration of cadmium in the hyperaccumulator plant species *Sedum alfredii*. *Plant Physiol.* 157 1914–1925. 10.1104/pp.111.183947 22025609 PMC3327216

[B69] Tian S. Lu L. Xie R. Zhang M. Jernstedt J. A. Hou D. (2015). Supplemental macronutrients and microbial fermentation products improve the uptake and transport of foliar applied zinc in sunflower (*Helianthus annuus* L.) plants. Studies utilizing micro X-ray florescence. *Front. Plant Sci.* 5:808. 10.3389/fpls.2014.00808 25653663 PMC4300865

[B70] Tian S. Lu L. Yang X. Webb S. M. Du Y. Brown P. H. (2010). Spatial imaging and speciation of lead in the accumulator plant sedum alfredii by microscopically focused synchrotron x-ray investigation. *Environ. Sci. Technol.* 44 5920–5926. 10.1021/es903921t 20608726

[B71] Tsuji K. Injuk J. Van Grieken R. (2005). *X-Ray Spectrometry: Recent Technological Advances.* Hoboken, NJ: John Wiley & Sons.

[B72] Van Grieken R. E. Markowicz A. A. (1993). *Handbook of X-ray Spectrometry: Methods and Techniques.* New York, NY: Marcel Dekker Incorporated.

[B73] von Wiren N. Marschner H. Romheld V. (1995). Uptake kinetics of iron-phytosiderophores in two maize genotypes differing in iron efficiency. *Physiol. Plant.* 93 611–616. 10.1034/j.1399-3054.1995.930405.x

[B74] Xiong Z. T. (1998). Lead uptake and effects on seed germination and plant growth in a Pb hyperaccumulator *Brassica pekinensis* Rupr. *Bull. Environ. Contam. Toxicol.* 60 285–291. 10.1007/s001289900623 9470991

[B75] Yanqun Z. Yuan L. Schvartz C. Langlade L. Fan L. (2004). Accumulation of Pb, Cd, Cu and Zn in plants and hyperaccumulator choice in Lanping lead-zinc mine area, China. *Environ. Int.* 30 567–576. 10.1016/j.envint.2003.10.012 15031017

[B76] Zaman Q. Aslam Z. Yaseen M. Ihsan M. Z. Khaliq A. Fahad S. (2018). Zinc biofortification in rice: leveraging agriculture to moderate hidden hunger in developing countries. *Arch. Agron. Soil Sci.* 64 147–161. 10.1080/03650340.2017.1338343

