# Supplementary material for: Laboratory Microprobe X-Ray Fluorescence in Plant Science: Emerging Applications and Case Studies
Source: Front Plant Sci. 2018 Nov 14;9:1588. doi: 10.3389/fpls.2018.01588 (PMC6246888; doi:10.3389/fpls.2018.01588)
Supplement: Supplementary file 1 [file Data_Sheet_1.pdf]

Supplementary Material

Laboratory microprobe X-ray fluorescence in plant science: Emerging applications and case studies

Eduardo S. Rodrigues[1], Marcos H. F. Gomes[1], Nádia M. Duran[1], João G. B. Cassanji[1], Tatiana N. M. da Cruz[1], Analder Sant'Anna Neto[2], Susilaine M. Savassa[1], Eduardo de Almeida[1] and Hudson W. P. Carvalho*[1]

*** Correspondence:**
Hudson W. P. Carvalho
hudson@cena.usp.br

1 Supplementary Figures and Tables

1.1 Supplementary Figures

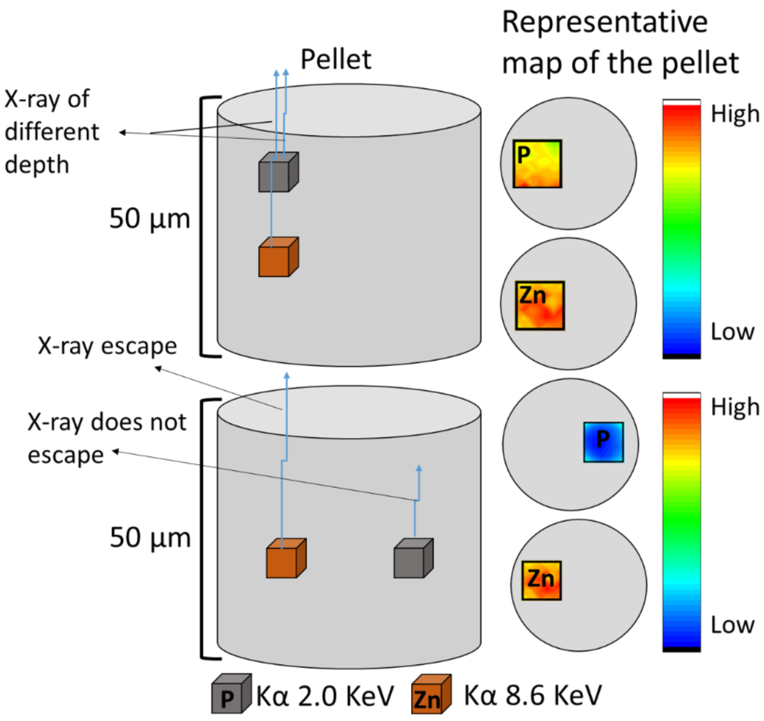

Supplementary Figure 1. Representative figure to demonstrate that the phosphorus and zinc do not occupy the same space region when it plots a 2D map

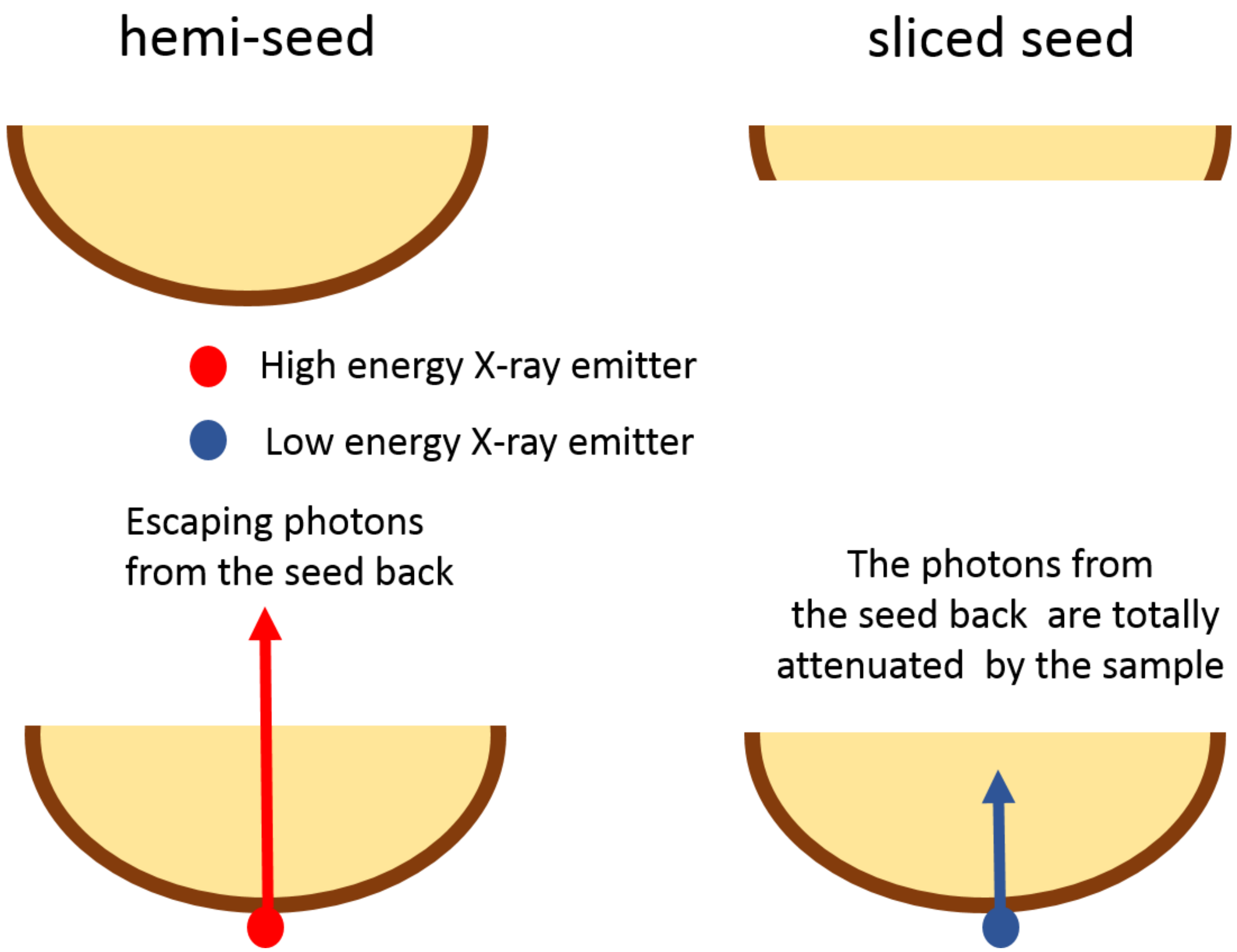

Supplementary Figure 2. On the top the schema shows two ways of preparing the seed sample for measurement. One can work with the hemi-seed, or slice it to obtain a thinner section. The thickness of a *Phaseolus vulgaris* hemi-seed may vary from *ca*. 2 to 4 mm. For nutrients such as phosphorus, sulfur, K and Ca this is an infinitely thick sample. However, for heavier elements such Zn the sample becomes intermediate thick, whereas for molybdenum it is nearly infinitely thin sample. On the bottom the Figure shows that depending on the element, the XRF detector may count photons coming from primed nutrients stuck on the back seed coat. In this case, the 2D projection would be misleading since one could conclude that the primed nutrients diffused inside of the cotyledon. For light elements, or certain L and M edges of heavier elements this effect cannot happen because of the high attenuation coefficient of the sample. One way to circumvent it consists in slicing the sample removing the back seed coat.

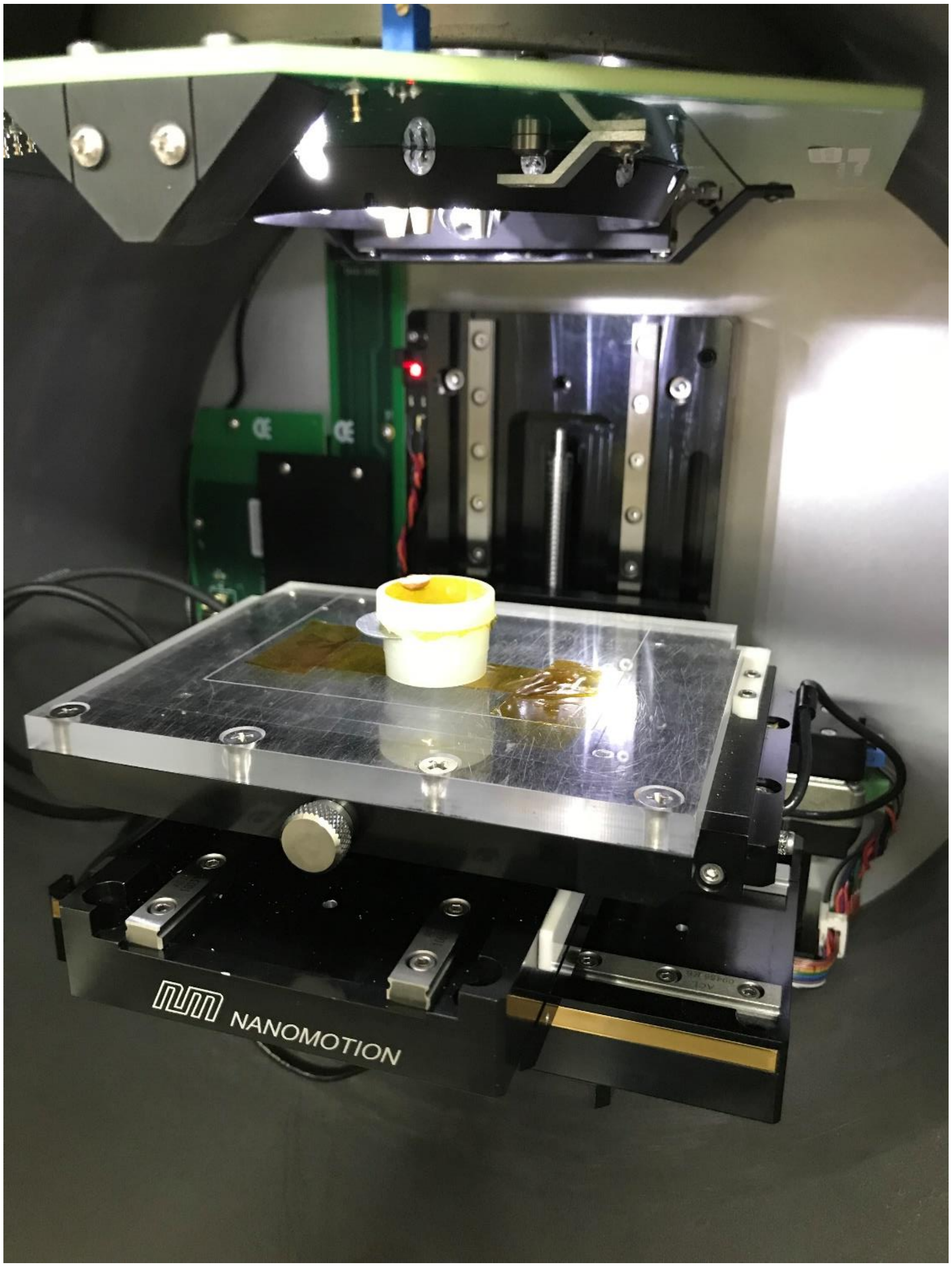

Supplementary Figure 3. µ-XRF experimental setup used for Zn mapping in a common bean seed

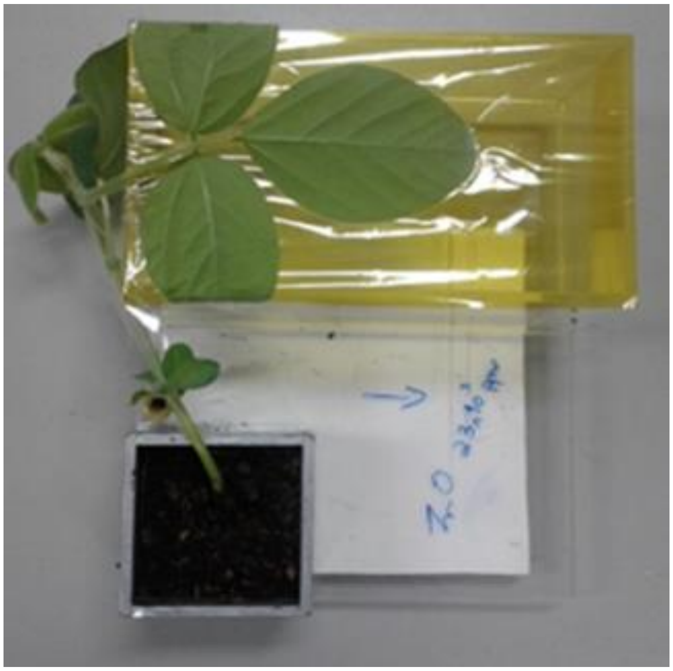

Supplementary Figure 4. Sample holder designed for mapping *in vivo* soy bean leaf. The plants were fixed on a Kapton film maintaining the leaf surface stretched for the analysis

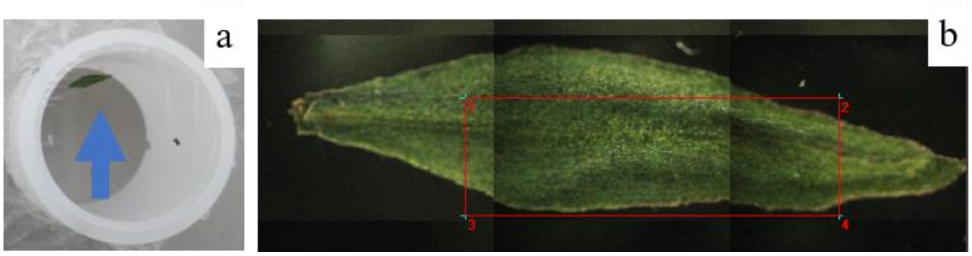

Supplementary Figure 5. (a) Sample holder used for fixed the *Eucalyptus urophylla* x *E. grandis* leaf. Arrow blue indicating the sample leaf. (b) Selected area (red rectangle) of the above-mentioned leaf for elemental mapping by micro-XRF.

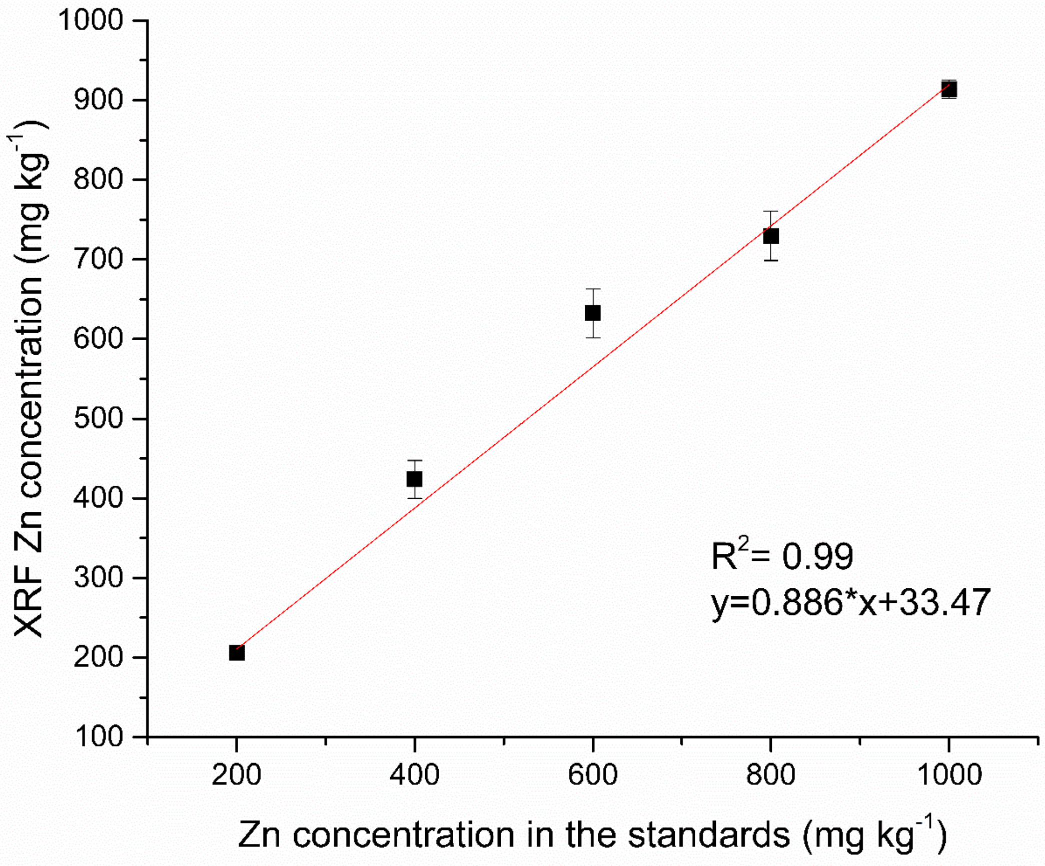

Supplementary Figure 6. XRF Zn concentration (mg kg^{-1}) using the XRF proposed method as function of its concentration (mg kg^{-1}) in the cellulose pellet standards.

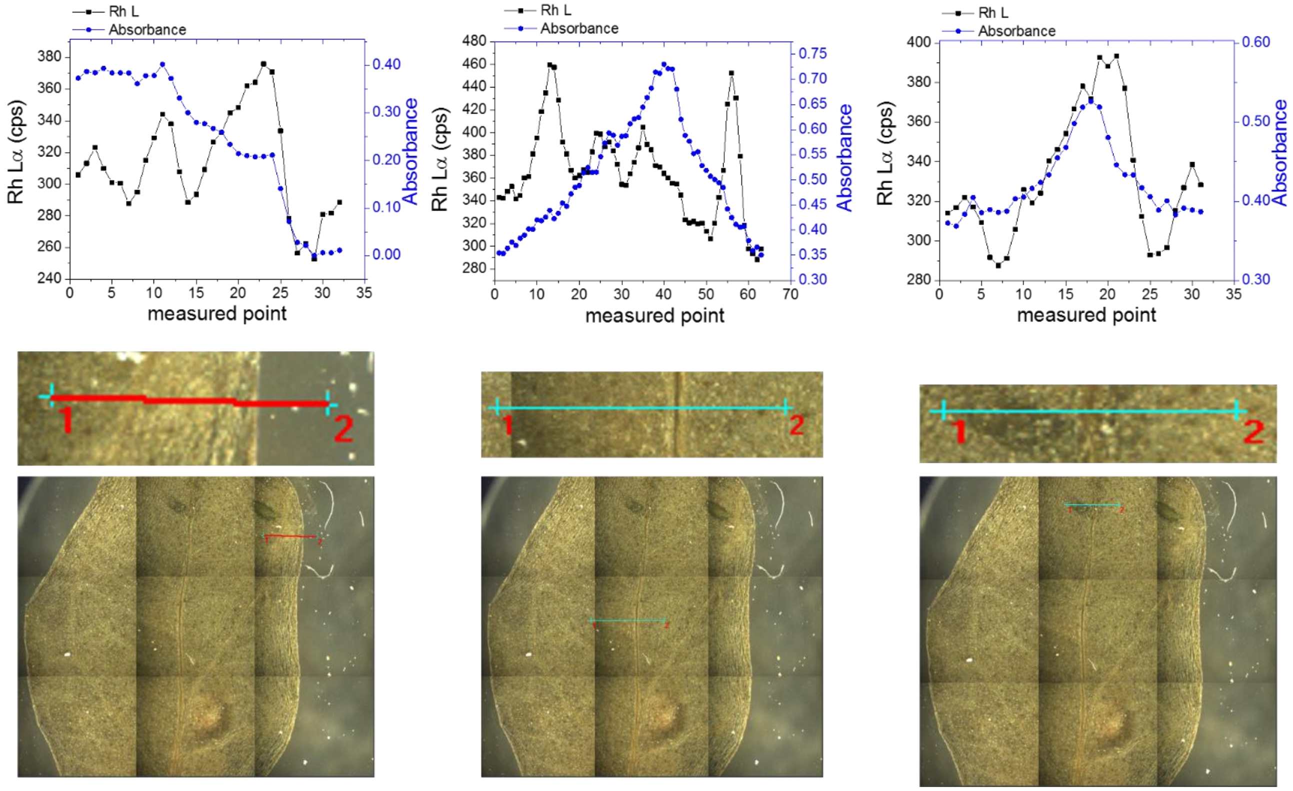

Supplementary Figure 7. The difference between the thickness of the central vein and leaf blade was estimated by monitoring the value of the absorbance (A). Since $A = c\ \mu\ x$, where c is concentration of the absorber in mass per volume, µ molar absorption coefficient and x is the sample optical length, one can relatively estimate the sample thickness by the determining. Several measurements indicated that the main vein presents nearly twice as thick as the blade. The Rh L scattering peak did not present a good correlation with the sample thickness, further studies must be carried out to investigate the behavior or Rh K Compton peaks.
